# Identification of Compound Heterozygous Variants in LRP4 Demonstrates That a Pathogenic Variant outside the Third β-Propeller Domain Can Cause Sclerosteosis

**DOI:** 10.3390/genes13010080

**Published:** 2021-12-28

**Authors:** Yentl Huybrechts, Eveline Boudin, Gretl Hendrickx, Ellen Steenackers, Neveen Hamdy, Geert Mortier, Guillermo Martínez Díaz-Guerra, Milagros Sierra Bracamonte, Natasha M. Appelman-Dijkstra, Wim Van Hul

**Affiliations:** 1 Center of Medical Genetics, University of Antwerp and University Hospital Antwerp, 2650 Antwerp, Belgium; yentl.huybrechts@uantwerpen.be (Y.H.); eveline.boudin@uantwerpen.be (E.B.); gretl.hendrickx@uantwerpen.be (G.H.); ellen.steenackers@uantwerpen.be (E.S.); geert.mortier@uantwerpen.be (G.M.); 2 Department of Internal Medicine, Division Endocrinology, Leiden University Medical Center, 2332 ZA Leiden, The Netherlands; n.a.t.hamdy.2@umail.leidenuniv.nl (N.H.); N.M.Appelman-Dijkstra@lumc.nl (N.M.A.-D.); 3 Endocrinology and Nutrition Resident, 12 de Octubre University Hospital, 28041 Madrid, Spain; guillermo.martinez@salud.madrid.org (G.M.D.-G.); milagrossierra1@gmail.com (M.S.B.)

**Keywords:** sclerosteosis, canonical WNT signaling, LRP4, SOST, mutation analysis, rare bone disease

## Abstract

Sclerosteosis is a high bone mass disorder, caused by pathogenic variants in the genes encoding sclerostin or LRP4. Both proteins form a complex that strongly inhibits canonical WNT signaling activity, a pathway of major importance in bone formation. So far, all reported disease-causing variants are located in the third β-propeller domain of LRP4, which is essential for the interaction with sclerostin. Here, we report the identification of two compound heterozygous variants, a known p.Arg1170Gln and a novel p.Arg632His variant, in a patient with a sclerosteosis phenotype. Interestingly, the novel variant is located in the first β-propeller domain, which is known to be indispensable for the interaction with agrin. However, using luciferase reporter assays, we demonstrated that both the p.Arg1170Gln and the p.Arg632His variant in LRP4 reduced the inhibitory capacity of sclerostin on canonical WNT signaling activity. In conclusion, this study is the first to demonstrate that a pathogenic variant in the first β-propeller domain of LRP4 can contribute to the development of sclerosteosis, which broadens the mutational spectrum of the disorder.

## 1. Introduction

**Figure 1:**
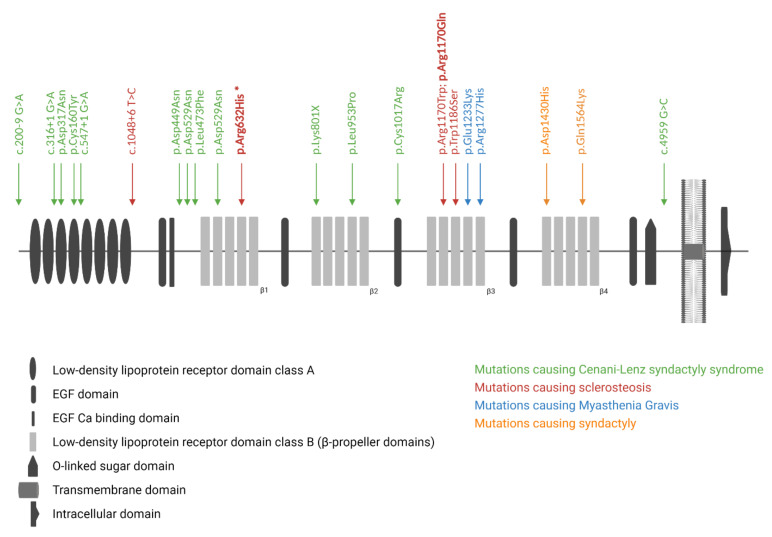
Overview of the protein structure of LRP4, and the reported pathogenic variants in LRP4-related disorders. Disease-causing variants for sclerosteosis, isolated syndactyly, Cenani–Lenz syndactyly syndrome, and myasthenia gravis are shown, as well as the newly identified p.Arg632His variant (indicated by the asterisk) in the first β-propeller (β1) domain of LRP4.

Sclerosteosis (OMIM #269500; #614305) is a rare sclerosing bone disorder which is marked by progressive hyperostosis, most prominently affecting the skull, mandible, and tubular bones [1,2]. In addition, patients with sclerosteosis often present with syndactyly of the fingers and toes, and a relatively tall stature. Due to progressive bone growth of the skull base, patients often suffer from facial paralysis, hearing loss, and blindness due to compression of the cranial nerves. Furthermore, the increased bone mass of the skull can also lead to increased intracranial pressure, resulting in headaches and even sudden death in some cases. The identification of homozygous loss-of-function variants in *SOST*, which encodes sclerostin, as the genetic cause for sclerosteosis was an important breakthrough in the bone field [3,4,5]. Subsequent studies demonstrated that sclerostin, which is mainly expressed by the osteocytes, is a major regulator of bone formation as an antagonist of the canonical WNT signaling pathway [6,7]. However, not all sclerosteosis cases are caused by variants in *SOST*. Ten years after the identification of *SOST* as the disease-causing gene, pathogenic variants in *LRP4*, which encodes the low-density lipoprotein receptor-related protein 4 (LRP4, Figure 1), were identified in *SOST*-negative sclerosteosis patients [8,9]. Studies demonstrated that LRP4 can interact with sclerostin, and facilitate the inhibitory actions of sclerostin on the canonical WNT signaling pathway, and, consequently, affect bone formation [8,10,11,12]. So far, we and others were able to identify three sclerosteosis-causing variants in *LRP4* (p.Trp1186Ser, p.Arg1170Trp, p.Arg1170Gln) [8,13,14]. All these variants are located in the cavity of the third β-propeller domain [13,15]. This region was also shown to be essential for the interaction of sclerostin with LRP4, resulting in increased canonical WNT signaling activity and bone formation when this binding is impaired [15]. Recently, the importance of the third β-propeller domain has been emphasized by the successful generation of two novel mouse models, the Lrp4 Arg1170Gln knock-in mouse and the Lrp4 Arg1170Trp knock-in mouse, both recapitulating the sclerosteosis phenotype observed in human patients [16,17]. However, more recently, Bukowska-Olech et al. reported on a homozygous splice-site variant (c.1048 + 6 T > C), located before the β-propeller domains of LRP4, in a sclerosteosis patient. As no functional studies were performed, it is unclear whether this identified variant leads to a complete loss of function, and whether it also affects the sclerostin–LRP4 interaction [18]. In addition to the aforementioned variants in the third β-propeller domain, genetic variation spread across other regions of *LRP4* is shown to cause other disorders (Figure 1), such as Cenani–Lenz syndactyly syndrome (OMIM #212780), myasthenia gravis (OMIM #616304), and isolated syndactyly [15,19,20]. Some of these variants are also shown to affect canonical WNT signaling activity; however, not via disrupting the binding of LRP4 with sclerostin, but due to a reduced expression at the cell membrane [13].

Based on the published data, it seemed clear that pathogenic variants in *LRP4* can result in distinct phenotypes, depending on the location of these variants. In the current study, we performed a mutation analysis of the *SOST* and *LRP4* genes in a sclerosteosis patient, identifying compound heterozygous variants in the first and third β-propeller domain of *LRP4*. Finally, to investigate the functional effect of these variants on canonical WNT signaling, luciferase reporter assays in an osteoblast-like cell line were performed.

## 2. Materials and Methods

### 2.1. Patient Material

Blood and bone biopsy samples were obtained after informed consent of the patient. Genomic DNA was isolated from peripheral blood from the patient and his parents using standard techniques. Transiliac bone biopsies were performed with a Bordier trephine (8.0 mm internal diameter) following double fluorochrome labeling with 200 mg tetracycline HCl, according to a schedule of 2 days on, 10 days off, and 2 days on. Five to seven days after the last tetracycline dose, the biopsy was taken and stored in 70% ethanol, and embedded in methyl methacrylate at Inserm UMR 1033, Lyon, France [21].

### 2.2. Mutation Analysis

All exons and intron/exon boundaries of the *SOST* and *LRP4* genes were analyzed using Sanger sequencing as previously described [13]. Primer sequences are available upon request. In short, amplification of all amplicons was performed using GoTaq2 polymerase (Promega Corporation, Madison, WI, USA) according to the supplier’s protocol. Afterwards, primers and unincorporated dNTPs were removed using exonuclease I (New England Biolabs, Ipswich, MA, USA) and calf intestine alkaline phosphatase (CIAP, Roche Applied Science, Hoffmann–La Roche AG, Basel, Switzerland). Finally, a sequencing reaction was performed directly on the purified fragments with the ABI 310 Genetic Analyzer (Applied Biosystems, Foster City, CA, USA), using the ABI Prism BigDye Terminator Cycle Sequencing Ready Reaction Kit, version 1.1 (Applied Biosystems, Foster City, CA, USA).

### 2.3. Expression Constructs and In Vitro Mutagenesis

An expression construct containing the human full-length *LRP4* coding sequence (corresponding with the ENST00000378623 transcript in the Ensembl database; http://www.ensembl.org/index.html (accessed on 15 April 2019)) was kindly provided by Michaela Kneissel (Novartis Pharma AG, Basel, Switzerland) [8]. Both variants, i.e., c.3509G > A (p.Arg1170Gln) and c.1895G > A (p.Arg632His), were introduced in the full-length wild-type construct using the QuickChange II XL Site-Directed Mutagenesis Kit (Agilent, Santa Clara, CA, USA). Primers were designed using the QuickChange Primer Design tool (https://www.agilent.com/store/primerDesignProgram.jsp (accessed on 7 June 2019)). Primer sequences are available upon request. The complete insert sequence was verified for the presence of the variant and the absence of PCR errors by direct DNA sequencing. Remaining expression constructs were obtained as described previously [22].

### 2.4. Luciferase Reporter Assay

The SaOS-2 cell line was grown in Dulbecco’s Modified Eagle Medium (DMEM, Thermo Fisher Scientific, Waltham, MA, USA) supplemented with fetal bovine serum (FBS, 10% *v*/*v*, Thermo Fisher Scientific, Waltham, MA, USA) and penicillin/streptomycin (1% *v*/*v*, Thermo Fisher Scientific, Waltham, MA, USA). Twenty-four hours before transfection, cells were plated at 0.3 × 10^5^ cells/well in 96-well plates. Cells were transfected using ViaFect™ (Promega Corporation, Madison, WI, USA) according to the manufacturer’s instructions. WNT1-V5 (40 ng), mesdc-2 (8 ng), LRP5 (8 ng), pRL-TK (2 ng), and TOPFlash (40 ng) constructs were co-transfected with WT or different mutant LRP4 constructs (8 ng). Depending on the experiment, different amounts of HA-mSost (30 ng and 60 ng) were co-transfected. When needed, empty pcDNA3.1 vector was added to make the total DNA amount equal for all conditions. Each transfection was carried out in triplicate and repeated independently in three separate experiments. Forty-eight hours after transfection, cells were lysed, and firefly and renilla luciferase activity were measured on a Glomax Multi + Luminometer (Turner Designs, Sunnyvale, CA, USA) using the Dual-Luciferase Reporter Assay System (Promega Corporation, Madison, WI, USA) according to manufacturer’s instructions. Ratios of the firefly and renilla luciferase measurements were calculated and expressed as relative to a negative control.

### 2.5. Statistical Analysis

Results are shown as mean values ± standard deviation (SD), and statistical analysis is performed using Student’s *t*-tests (SPSS 20.0 software package, SPSS Inc., Chicago, IL, USA). A *p*-value < 0.05 was considered statistically significant.

## 3. Results

### 3.1. Clinical Description

The proband is a 57-year-old Spanish man who was born from healthy parents, and who has three healthy brothers. He presented with lower neuron facial palsy at the left site at the age of 6 years. More than ten years later, bilateral compression of the auditory nerves due to sclerosis resulted in impaired hearing. These findings were followed by recurrent headaches and dizzy spells. At that time, he was diagnosed with Van Buchem disease. The patient suffered from episodes of tonic-clonic seizures, his deafness progressed further, and he needed hearing aids. An MRI indicated osseous encroachment of the posterior fossa, and reduction of the cisternal space at the level of the foramen magnum. When the patient was 38 years old, he again visited the clinic, as he continued to have headaches, seizures, dizzy spells, and his hearing loss worsened. At that time, his height was 180 cm. He had a large skull with prominent mandible, and showed facial asymmetry due to the facial nerve palsy. He was wearing hearing aids, showed bilateral exophthalmos (left > right), but had a normal visual field with no evidence of optic atrophy or papilledema. His hands showed soft tissue webbing of the third and fourth digits. Biochemical analysis showed no abnormalities in renal, liver, gonadal, and thyroid function. In urine, normal calcium and hydroxyproline levels were measured. Moreover, serum levels of calcium (corrected for albumin), phosphate, magnesium, alkaline phosphatase, osteocalcin, parathyroid hormone, and vitamin D were within normal reference ranges. Elevated serum sclerostin levels were measured (1.7 ng/mL (0.64 ng/mL ± 0.15), TECO immunoassay).

**Figure 2:**
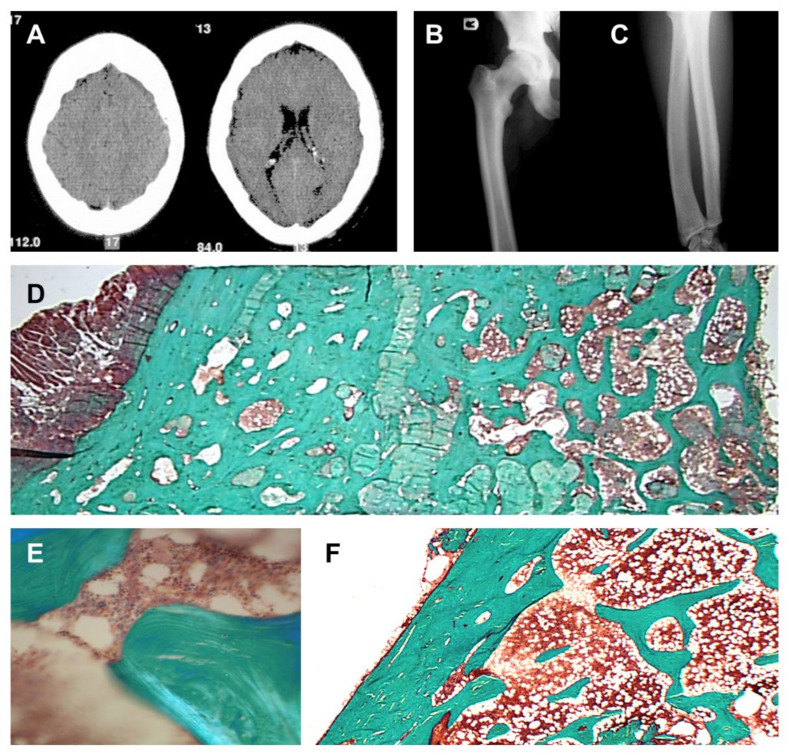
Bone phenotype in the proband. (**A**) CT scan of the patient’s skull, demonstrating a thickened calvarium. (**B**,**C**) Radiograph showing endosteal cortical hyperostosis of the femur (left), and radius and ulna (right). (**D**) Bone biopsy showing very high cancellous bone volume, no evidence of an increased osteoid volume, and the absence of active osteoblasts on the bone surface. Magnification 10× (insert (**E**), magnification 125×). (**F**) Bone biopsy of a healthy control demonstrating a clear distinction between the cortical and trabecular bone compartment, and normal bone volumes.

Imaging studies demonstrated sclerosis of the axial skeleton, and endosteal cortical hyperostosis of the long bones (Figure 2A–C). DXA measurements demonstrated a highly increased bone mineral density of the femoral neck (Z-score: +7.83 and +7.94, respectively, left and right) and lumbar spine (Z-score: +8.46). In addition, a bone biopsy demonstrated no evidence for increased osteoid surfaces or volumes, and the absence of active osteoblasts on the bone surface (Figure 2D–F). Sclerostin expression was observed in the osteocytes (data not shown). Finally, histomorphometric analysis demonstrated massive well-connected trabeculae, very high cancellous bone volume (51.9% (19.5% ± 4.9)), marginally increased eroded surfaces (4.9% (3.6% ± 1.1)), and a slightly increased mineralization rate (1.18 µm/day (0.72 ± 0.12)).

### 3.2. Genetic Screening of SOST and LRP4

**Figure 3:**
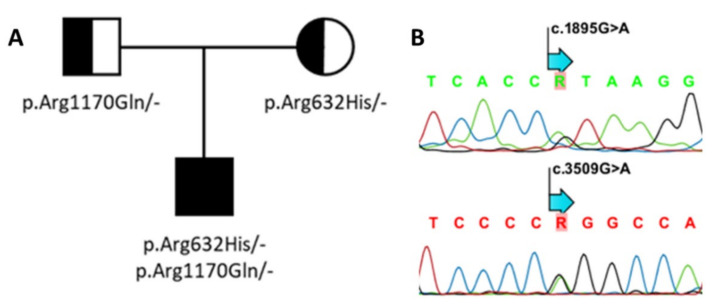
Genetic analysis of *LRP4*. (**A**) Family pedigree. (**B**) Using Sanger sequencing, two compound heterozygous variants—p.Arg632His; c.1895G > A (above) and p.Arg1170Gln; c.3509G > A (below)—were identified in the proband.

As the patient was thought to suffer from sclerosteosis, *SOST* and *LRP4* were analyzed for putative variants. Genetic screening was initially focused on the exonic regions covering the third β-propeller domain of *LRP4*. This revealed the heterozygous presence of the known p.Arg1170Gln (c.3509G > A, exon 25) variant inherited from the father (Figure 3). A deleterious effect of this variant was initially supported by the in silico analysis (CADD-score v1.5: 28.3; MutationTaster: disease-causing, score 0.9; PolyPhen-2: probably damaging, score 1). The homozygous presence of this variant was previously reported by us in a patient with sclerosteosis, and shown to affect canonical WNT signaling in vitro [13]. Thereafter, the other coding regions of *LRP4* were also screened for possible pathogenic variants, which resulted in the identification of a second heterozygous base pair change (c.1895G > A, exon 14, p.Arg632His), which was inherited from the mother, and absent in gnomAD (v3.1.1; Figure 3). This variant is located in the first β-propeller domain of LPR4, and in silico analyses predicted that the variant is likely to be disease-causing (CADD-score v1.5: 28; MutationTaster: disease-causing, score 0.9; PolyPhen-2: probably damaging, score 1).

### 3.3. Functional Evaluation of p.Arg632His LRP4

**Figure 4:**
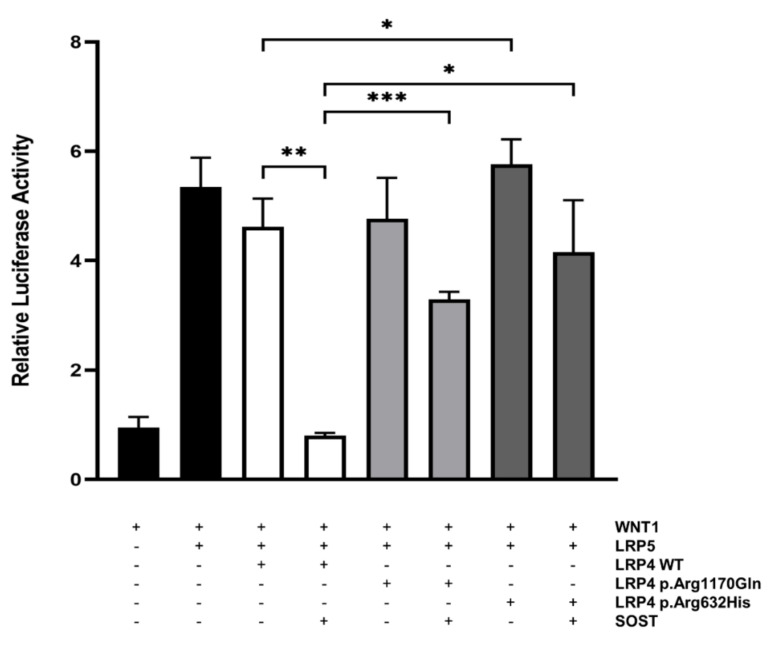
Functional evaluation of wild-type and mutant forms of LRP4 in SaOS-2 cells. SaOS-2 cells were transiently transfected with WNT1 and LRP5 to activate canonical WNT signaling. To investigate and compare their inhibitory actions on this pathway, wild-type (WT) and mutant (p.Arg1170Gln or p.Arg632His) forms of LRP4 were co-transfected with or without SOST. Luciferase activity is expressed as relative to a negative control. Bars represent mean values ± SD. * *p* < 0.05; ** *p* < 0.01; *** *p* < 0.001.

In order to investigate the inhibitory effect of the p.Arg632His LRP4 missense variant on canonical WNT signaling, we performed a WNT-luciferase reporter experiment (TOPFlash) in SaOS-2 cells, an osteoblast-like cell line. Parallel transfection of the previously reported LRP4 mutant (p.Arg1170Gln) was used as a control. We demonstrated that in the presence of the p.Arg632His variant in LRP4, the inhibitory capacity of sclerostin on the canonical WNT signaling activity was significantly impaired, to a similar extent as the reported p.Arg1170Gln LRP4 mutant (Figure 4). Even more, transfection of p.Arg632His LRP4 alone results in a significant increase of canonical WNT signaling activity, compared to the transfection of wild-type LRP4. Overall, these results support a causative effect of the novel p.Arg632His LRP4 mutant.

## 4. Discussion

Our study shows the identification and functional investigation of two compound heterozygous variants in *LRP4* in a Spanish sclerosteosis patient (Figure 3). One of the two variants, i.e., p.Arg1170Gln in the third β-propeller domain, has previously been reported by us in a homozygous state as being disease-causing for sclerosteosis in a patient of Portuguese descent [13]. Interestingly, the other heterozygous variant (p.Arg632His) has not been reported before, and is located outside the region in *LRP4* that is usually mutationally affected in sclerosteosis patients. Functional investigation of both variants in parallel demonstrated that the presence of the p.Arg632His variant impaired the inhibitory capacity of sclerostin on canonical WNT signaling activity, similar to the p.Arg1170Gln variant (Figure 4).

In the past decade, the LRP4 receptor became a well-known facilitator of the sclerostin-dependent inhibition of canonical WNT signaling, a major pathway in the osteoblast-mediated bone formation process. This was evidenced by the identification of disease-causing variants in *LRP4* or *SOST* in sclerosteosis patients, a severe sclerosing bone disorder specifically characterized by progressive bone overgrowth [8,13,14]. In addition, LRP4 is involved in maintaining the neuromuscular junction (NMJ) via interaction with agrin, and via formation of the muscle-specific kinase (MuSK) signaling complex [15,23,24]. As shown by Ohkawara et al., the modulation of the different pathways is dependent on specific regions in the third β-propeller domain of LRP4. More specifically, canonical WNT signaling activity is linked to the cavity of this domain, whereas the edges of this domain mediate MuSK signaling, which explains the distinct phenotypes caused by pathogenic variants in different regions of the third β-propeller domain [15].

So far, all previously reported sclerosteosis-causing variants are located in the highly conserved cavity of the third β-propeller domain, which is an essential region for the interaction with sclerostin [8,13,14]. Besides sclerosteosis, other LRP4-related disorders have been described, in which the disease-causing variants are located outside this specific region of the LRP4 protein (Figure 1). Cenani–Lenz syndactyly syndrome and isolated syndactyly are caused by pathogenic variants outside the third β-propeller domain [19,20], and myasthenia gravis is associated with pathogenic variants in the edge of this domain [15]. In this regard, the identification of the novel p.Arg632His variant in LRP4 in the first β-propeller domain is noteworthy, since our functional studies have demonstrated that the inhibitory action of sclerostin is clearly disturbed (Figure 4). Although this domain was described as indispensable for the interaction with agrin [23], the patient’s phenotype points towards a direct effect on the canonical WNT signaling pathway. Hence, the question remains whether there is a location-specific effect, as previously reported for the third β-propeller domain. Another possibility is a tissue-specific effect, depending on the availability of WNT signaling components in the microenvironment, e.g., sclerostin in bone tissue. Although additional research is required to elucidate the underlying mechanism leading to this reduced canonical WNT signaling activity, it might also be that the p.Arg632His variant causes impaired expression of LRP4 on the cell membrane, similar to what was previously reported for *LRP4* variants in the first β-propeller domain causing Cenani–Lenz syndrome [19].

In conclusion, we report the identification of two compound heterozygous variants, p.Arg1170Gln and p.Arg632His, in *LRP4* in a patient with a sclerosteosis phenotype. To our knowledge, the p.Arg632His variant is the first variant described in the first β-propeller domain of LRP4 that contributes to the development of a sclerosteosis phenotype, thereby expanding the mutational spectrum of the disorder, which is important for future diagnostic testing.

## Data Availability

The data presented in this study are available on request from the corresponding author.
